# Blockade of Mast Cell Activation Reduces Cutaneous Scar Formation

**DOI:** 10.1371/journal.pone.0085226

**Published:** 2014-01-22

**Authors:** Lin Chen, Megan E. Schrementi, Matthew J. Ranzer, Traci A. Wilgus, Luisa A. DiPietro

**Affiliations:** Center for Wound Healing and Tissue Regeneration, University of Illinois at Chicago, Chicago, Illinois, United States of America; Albert Einstein College of Medicine, United States of America

## Abstract

Damage to the skin initiates a cascade of well-orchestrated events that ultimately leads to repair of the wound. The inflammatory response is key to wound healing both through preventing infection and stimulating proliferation and remodeling of the skin. Mast cells within the tissue are one of the first immune cells to respond to trauma, and upon activation they release pro-inflammatory molecules to initiate recruitment of leukocytes and promote a vascular response in the tissue. Additionally, mast cells stimulate collagen synthesis by dermal fibroblasts, suggesting they may also influence scar formation. To examine the contribution of mast cells in tissue repair, we determined the effects the mast cell inhibitor, disodium cromoglycate (DSCG), on several parameters of dermal repair including, inflammation, re-epithelialization, collagen fiber organization, collagen ultrastructure, scar width and wound breaking strength. Mice treated with DSCG had significantly reduced levels of the inflammatory cytokines IL-1α, IL-1β, and CXCL1. Although DSCG treatment reduced the production of inflammatory mediators, the rate of re-epithelialization was not affected. Compared to control, inhibition of mast cell activity caused a significant decrease in scar width along with accelerated collagen re-organization. Despite the reduced scar width, DSCG treatment did not affect the breaking strength of the healed tissue. Tryptase β1 exclusively produced by mast cells was found to increase significantly in the course of wound healing. However, DSCG treatment did not change its level in the wounds. These results indicate that blockade of mast cell activation reduces scar formation and inflammation without further weakening the healed wound.

## Introduction

Mast cells have long been regarded primarily as effector cells in hypersensitivity reactions. While their importance during an allergic reaction cannot be denied, mast cells also have a significant influence on the tissue repair process [1]–[3]. Mast cells are widely distributed in the body, and are prominent near surfaces exposed to the environment, including the skin [1]. Thus, mast cells are one of the first cells to respond to trauma and stimulate an immune response through the release of preformed biological mediators. In response to wounding, mast cells degranulate and the granule contents stimulate activation and proliferation of endothelial cells within the tissue [1], [4], [5]. Cytokines released by mast cells promote proinflammatory cytokine production by resident cells, attracting inflammatory cells. The release of vasoactive amines stimulates vessel permeabilization, promoting the influx of neutrophils, macrophages and additional mast cells into the tissue. Thus the activation and degranulation of resident mast cells intensifies and extends the inflammatory response [1], [4]. Mast cells are prominent in scar tissue, and activated mast cells remain in the scar up to a year after wounding [6]. Mast cell activation may influence wound remodeling as excess inflammation and cytokine production can promote scar formation [2], [4], [6]. Scarless healing of oral mucosal wounds in the red Duroc pig, a common model for hypertrophic scarring, has less numbers of mast cells compared to skin wounds [7]. Mast cells may also more directly influence collagen synthesis. Within the dermis, activated mast cells release mediators, including tryptase, that stimulate fibroblasts to synthesize collagen [1], [8]–[10]. Tryptase has also been reported to induce the differentiation of fibroblasts into myofibroblasts [10]. Myofibroblasts play a fundamental role in wound contraction, a process that reduces wound volume and speeds closure [11]. Moreover, myofibroblast activity has been linked to hypertrophic scarring and other fibrotic pathologies [12]. A commercially available mast cell stabilizer, ketotofen, reduced fibrosis and scarring in red duroc pigs [13]. In a scald injury model of WBB6F1-kit^w^/kit^w-v^ mast cell deficient mice, the dermis was thinner and fibrous proliferation was less extensive at the wound edge after inducing injury than in wild type mice [14]. Together, these studies suggest that mast cells may influence scar formation.

Most previous studies of the role of mast cells in wound healing have utilized the WBB6F1-kit^w^/kit^w-v^ mast cell deficient mice [14]–[16]. Due to a c-kit mutation, these mice have a nearly complete deficiency of both mast cells and melanocytes [17], [18]. However, they also have several other abnormalities that might influence healing outcomes, such anemia [19]. While previous studies are somewhat conflicting, the wounds of WBB6F1-kitw/kitw-v mice have been described to have reduced numbers of neutrophils, yet to exhibit minimal defects in wound closure [15].

The goal of the current study was to directly examine the role of mast cells on scar formation in a normally healing wound. To avoid the confounding phenotype of genetically mutant mast cell deficient mice, we utilized a pharmacologic approach to inhibit mast cells. DSCG inhibits calcium-induced membrane permeability, preventing mast cell degranulation, and has an established function in the prophylactic treatment of allergic diseases [20]–[22]. Using a mouse model of excisional wounds, we found that DSCG treatment decreased scar formation and inflammation without reducing skin strength. These data point to a distinct role for mast cells in scar formation in skin repair, and suggest that mast cell inhibition could improve healing outcomes.

## Materials and Methods

### Animals and Wound models

All animal procedures were approved by the Loyola University and University of Illinois at Chicago Institutional Animal Care and Use Committees. Six- to eight-week old female Balb/c mice (Harlan, Inc, Indianapolis, IN) were anesthetized under isoflurane (Abbott Laboratories, Abbott Park, IL) inhalation. Two dermal wounds were placed on opposite sides of the midline at the scapula level using a 3 mm punch biopsy instrument (Acu-Punch, Acuderm Inc., Ft. Lauderdale, FL) as previously described [23]. At various intervals after injury, wound and surrounding tissues were removed with a biopsy punch for analysis.

### Real time PCR

Total RNA was extracted from TriZol lysates per the manufacturer's instruction and treated with DNase I (Invitrogen). cDNA was prepared from total RNA using a Retroscript kit (Invitrogen). Collagen I mRNA expression was determined by a StepOne plus real time PCR system (Applied Biosystem, Foster City, CA) using gene specific primers (forward: 5′-GGTATGCTTGATCTGTATCTGC-3′and reverse: 5′-AGTCCAGTTCTTCATTGCATT-3′) and SYBR green mater mix (Applied Biosystem). The relative quantity of collagen I mRNA in tryptase β1 treated and untreated cells was determined using 2^−ΔΔCt^ method. GAPDH was used for normalization. The primers for GAPDH were as previously published [24].

### DSCG treatment of mice

To assess the effects of mast cell degranulation on would repair; mice were injected intraperitoneally with DSCG (Sigma) or PBS. Thirty minutes prior to wounding, 200 µl of DSCG (160 mg/kg) or PBS was administered as previously described [25]. Additional doses were given at 24, 48 and 72 hours post-wounding.

### Quantification of mast cells

To determine the number of mast cells within the wound bed, individual wounds were embedded and frozen and 10 µm sections were prepared using a cryostat. Frozen sections were thawed and fixed for 1 hour in Carnoy's fixative (60% ethanol, 30% chloroform, and 10% glacial acetic acid). Sections were stained for 2 hours at room temperature with 0.5% toluidine blue (Sigma) in 0.5N HCl in PBS. Following dehydration and xylene clearing, cover slips were mounted on each slide with Cytoseal (Richard-Allan Scientific, Kalamazo, MI). Mounted sections were viewed under a 20× objective, the wound bed was delineated, and the number of mast cells within the center of the wound bed was determined. The total area of the wound bed was measured using Scion Image. Three sections per mouse were averaged. Wounds from five mice per treatment group were counted, and mast cell numbers were expressed as the total number of mast cells/area (mm^2^) of the wound sections.

### Analysis of wound re-epithelialization

The extent of re-epithelialization was measured by histomorphometric analysis of 5 µm tissue sections from the central portion of the wound that had been stained with hematoxylin and eosin. Using a standard ocular grid, the distance between the muscle edges, and the distance that the epithelium had traveled across the wound were measured. In order to determine the percent re-epithelialization, the following formula was used: % Re-epithelialization = (Distance covered by the epithelium/Distance between muscle edges) ×100.

### Inflammatory cytokine analysis

Cytokine analysis was performed using the Bio-Rad, Bio-Plex assay. Individual wounds were homogenized in 1 ml cell lysis buffer (from the Cell Lysis Kit, Bio-Rad) containing 500 mM phenylmethylsulfonyl fluoride in dimethyl sulphoxide (both from Sigma). Tissue was further disrupted by sonication. Samples were centrifuged and supernatants filtered through a 32 mm syringe filter with 1.2 µm membrane (Pall Life Sciences, Cornwall, UK). The supernatants were collected and stored at −80°C. The Bio-Plex mouse cytokine assay for simultaneous quantitation of interleukin (IL)-1α, IL-1β, and CXCL1 was used according to the manufacturer's protocol. Briefly, the premixed standards were reconstituted in 0.5 ml cell lysis buffer, generating a stock concentration of 50 ng/ml for each cytokine. The standard stock was serially diluted in cell lysis buffer to generate 8 points for the standard curve. The assay was performed in a 96-well filtration plate supplied with the assay kit. Premixed beads (50 µl) coated with target capture antibodies were transferred to each well of the filter plate and washed twice with Bio-Plex wash buffer. Premixed standards or samples (50 µl) were added to each well containing washed beads. The plate was shaken for 30 seconds and then incubated at room temperature for 30 minutes with low-speed shaking. After incubation and washing, premixed detection antibodies (50 µl) were added to each well. The incubation was terminated after shaking for 10 minutes at room temperature. After washing three times, the beads were resuspended in 125 µl of Bio-Plex assay buffer. Beads were read on the Bio-Plex suspension array system, and the data is analyzed using Bio-Plex Manager™ software with 5PL curve fitting. Cytokine levels in picograms were normalized to total protein concentrations as determined by the Bio-Rad Protein Assay (Bio-Rad).

### Analysis of neutrophil myeloperoxidase (MPO)

Excisional wounds from DSCG treated and control animals (n = 6 per group, per time point) were harvested at 1, 2, and days post-injury, snap-frozen with liquid nitrogen and stored at −80°C until analysis. MPO levels were determined as previously described [26]. To prepare samples, individual wounds were homogenized in 2.0 mL of 20 mmol/L phosphate buffer, pH 7.4. Homogenates were centrifuged at 12,000× g for 45 minutes, and the supernatant was decanted. The pellets were resuspended in 1.0 mL of 50 mmol/L phosphate buffer containing 10 mmol/L ethylenediamine tetraacetic acid and 0.5% hexadecyltrimethylammonium bromide. After a freeze-thaw cycle, the samples were sonicated briefly and incubated a 60°C for 2 hour to release maximal MPO activity. The samples were centrifuged at 500 g for 10 minutes and the supernatant was transferred to 1.5 mL tubes for storage at −20°C.

For analysis, samples were thawed and MPO standard (Sigma) was diluted to generate a standard curve ranging from 0 to 3.0 units per mL. Then 12.5 µL aliquots of sample or standard were placed in 96-well tissue culture plates with 125 µL of assay buffer (0.1 mol/L phosphate buffer, pH 5.4, 1% hexadecyltrimethylammonium bromide, 0.43 mg per mL 3, 3′, 5, 5′- tetramethylbenzidine). The reactions were started by the addition of 12.5 µL of 15 mmol/L H_2_O_2_, incubated at 37°C for 15 minutes, and stopped with 250 µL cold 0.2 mol/L sodium acetate, pH 3.0. The absorbance of each sample was determined by reading absorbance at 655 nm within 10 minutes.

### Histological assessment of collagen content and architecture of scars

Wound samples from DSCG or PBS treated mice were harvested 14 and 21 days post-wounding. Skin samples were embedded in paraffin and 5 µm sections were cut, mounted on slides and stained with hematoxylin to verify a central wound location. To determine changes in the orientation of collagen fibers, tissue sections from DSCG or PBS treated mice were analyzed by picrosirius red staining (Sigma). Deparaffinized sections were hydrated, then sections were stained with a 0.01% solution of Sirius red F3BA in saturated aqueous picric acid (Electron Microscopy Sciences, Hatfield, PA) for 90 minutes. After staining, slides were washed for two minutes in 0.01 N HCl, rinsed for one minute in 80% ethanol, dehydrated, cleared and cover slips were mounted in Permount (Fisher, Fairlawn, NJ). The sections were examined under polarized light with a Zeiss AxioVert 200 microscope and images were captured at 20× magnification.

### Transmission electron microscopy (TEM)

For ultrastructural collagen analysis, normal skin and wound tissues were fixed in 4% glutaraldehyde. After rinsing with 0.1 mol/L sodium cacodylate buffer, samples were post-fixed in 1% osmium tetraxide, dehydrated in graded alcohols, and embedded in Spurr's epoxy resin (Electron Microscopy Sciences, Fort Washington, PA). Ultrathin sections (80 nm) were collected on grids, stained with uracyl acetate and lead citrate and examined using a Hitachi H-600 (75 kV) TEM as previously described [26]. The wound bed was identified using a serial section stained with toluidine blue. TEM images of collagen fibril cross-sections were taken at random within the boundaries of the wound bed at a magnification of 30,000× and the photographic negatives were scanned into Adobe Photoshop. The diameter of the collagen fibrils was measured using Scion Image. Five normal skin samples and five wound samples from each time point, all from different mice, were used for analysis. Each electron micrograph was divided into four parts for analysis, and fibrils from four micrographs per sample were measured. A total of 1100–2100 fibrils per animal were analyzed [26]. Images of fibroblasts within the wound bed were captured at 8000× and the photographs were scanned into Adobe Photoshop (Adobe Systems Incorporated, San Jose, CA).

### Measurement of fibril Density

The mean fibril density was determined by image analysis of TEM sections of wounds from either DSCG or PBS treated mice. Sections were analyzed by Scion Image software (Scion Corp., Frederick, MD). The area of each micrograph was determined using a freehand drawing tool. Each fibril was identified and the total area occupied by fibrils in each micrograph was counted using a colorization tool. Fibril density is expressed as the percent of total area occupied by fibrils from ten micrographs per mouse. At each time point, the percent area density was determined for ten micrographs from each of five mice.

### Measurement of scar width

Paraffin sections from the center of the wound bed were stained with Masson's trichrome stain (Sigma) as described [27]. The tissue was visualized using a Zeiss AxioVert 200 microscope and scar width was measured with a stage micrometer. The average scar width from three sections per mouse was calculated and a total of five mice per group were analyzed.

### Wound breaking strength

Under anesthesia, a 4 cm full thickness skin incision was made on the shaved backs of mice treated with PBS or DSCG. The incisions were closed with surgical clips, which were removed on the fifth post-operative day. On day 14 after surgery, animals were euthanized and three skin strips perpendicular to the incision per mouse were cut. These strips were subjected to tensiometry using a materials testing system, designed and built by the Department of Surgery and Instrument Models Facility at the University of Vermont [28]. Wound disruption strength was defined as the load required to break the wound along the incision and is represented in grams.

### Western Blot tryptase β1 analysis

To examine the protein levels of tryptase β1 in the wound tissues, normal skin tissues and wound samples were homogenized in RIPA buffer (Sigma) with protease inhibitors (Sigma). Samples were centrifuged at 13000 rpm at 4°C for 15 minutes, and protein concentration determined for the resulting supernatants. 150–180 µg µg of each protein sample was loaded onto a 10% Tris-glycine acrylamide gel (Bio-Rad). Separated proteins were transferred to a nitrocellulose membrane and first blocked with 5% skim milk in Tris buffered saline. Membranes were exposed to goat anti-mouse tryptase β1 at 0.2 µg/ml (R&D systems Minneapolis, MN) and a rabbit anti-human α-tubulin (Abcam, Cambridge, MA) at 1/3000 dilution which cross reacts with mouse α-tubulin for 1 hour at room temperature. The membrane was washed and then incubated with rabbit anti-goat and goat anti-rabbit HRP (Bio-Rad) at 1/2500 dilution. The membrane was developed using an ECL system (Piscataway, NJ). Imaging of the resulting membrane was performed and analyzed using a ChemiDoc system (Bio-Rad).

### Fibroblast cell culture

To investigate the effects of tryptase β1 on collagen I gene expression and the differentiation of dermal fibroblasts to myofibroblasts, dermal fibroblasts were isolated from mouse dorsal skin. To isolate fibroblasts, skin was first shaved and excised, and then the tissues were spread dermis side down in a sterile tissue culture dish and incubated overnight with 0.1% dispase at 4°C. Following removal of the epidermis, the dermis was cut into small pieces and incubated with 0.5% collagenase type I (Sigma) in DMEM at 37°C for 2 h, then dissociated into a cellular suspension. Cells were washed twice to remove collagenase then cultured in DMEM with 10% fetal bovine serum (FBS). The cells were incubated until 60–70% confluent and the medium was changed to DMEM without FBS for 24 hours. Cultures were treated with mouse recombinant tryptase β1 (R&D systems) at 1 µg/ml in the presence of the same concentration of heparin as a stabilizer of tryptase β1 [8]. Control cultures were treated with heparin only. At 24 and 48 hours, cells were harvested using TriZol for real time PCR analysis. At 72 hours, cells were fixed with acetone for 15 minutes and then subjected to immunofluorescent staining as described below.

### α-smooth muscle actin (SMA) immunofluorescent detection

Acetone fixed fibroblasts on chamber slides were incubated with 10% normal mouse serum for 45 minutes followed by FITC conjugated mouse monoclonal anti-α-SMA (Sigma) for 1 hour at room temperature. After washing, the slides were mounted with 50% glycerol in PBS, and then observed using a Zeiss AxioVert 200 fluorescence microscope. DAPI was used for nuclear staining. The images were recorded by an attached digital camera. 200 cells were counted in each slide, and the percent of α-SMA positively stained cells was calculated.

### Statistical analysis

Data were analyzed using GraphPad Prism (GraphPad Software, Inc., San Diego, CA). The mean and SEM were calculated for each data set. A two-way analysis of variance (ANOVA) followed by a Bonferroi post-test or t-test was performed. For studies with two groups, a t-test was used. For all analyses, values of p<0.05 were considered statistically significant.

## Results

### DSCG inhibits mast cell activation in wounds

**Figure 1:**
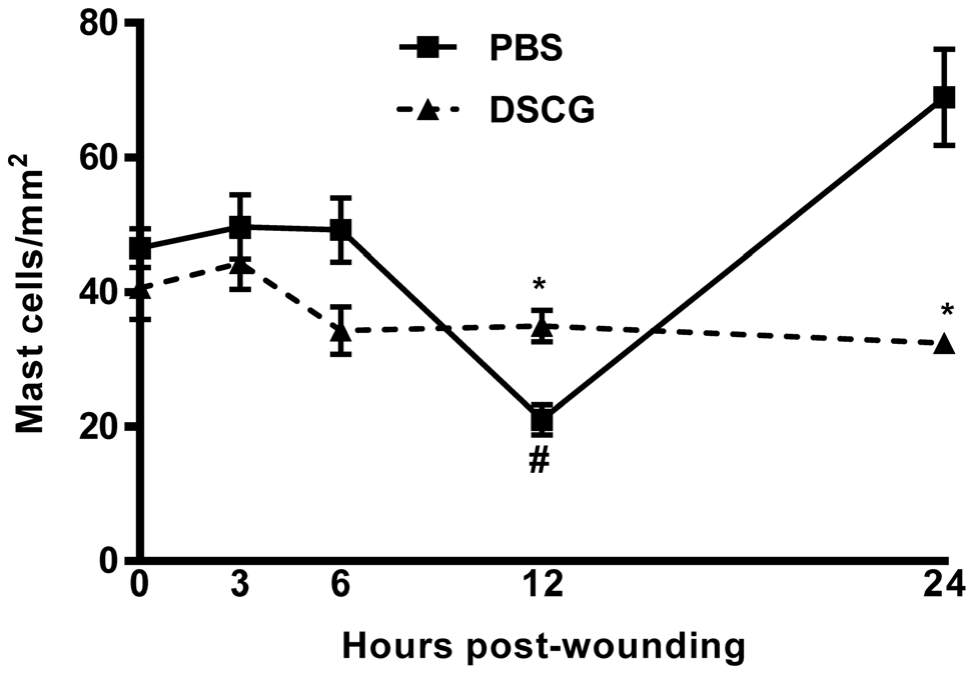
Quantitation of mast cell numbers after DSCG treatment. Total mast cells numbers were counted using toluidine blue staining of tissue sections from cutaneous wounds (squares) or wounds from animals treated with DSCG (triangles). The total number of mast cells were counted per wound, followed by measurement of total wound area. Three sections per mouse were averaged. Data is represented as total mast cells per area of the wound (n = 5). *p<0.05 compared to PBS group, #p<0.01 compared to 0 hour in PBS group by t-test.

The mast cell inhibitor, DSCG was used to assess the effects of mast cell activation on wound healing. To verify inhibition of mast cell activation after DSCG treatment, toluidine blue was used to stain mast cell granules. Fig. 1 shows that unwounded tissue from mice treated with DSCG and PBS contained similar numbers of mast cells. Upon wounding, the number of mast cells in PBS treated wounds decreased significantly by 12 hours (p<0.01), most likely representing massive degranulation. In contrast, the number of mast cells in DSCG treated wounds did not decrease compared to levels in normal tissues. Levels of visible mast cells in wounds of DSCG mice were significantly more than that in PBS treated wounds (p<0.05), again likely representing a relative decrease in degranulation due to DSCG treatment. In control mice, activation and loss of visible mast cells was followed by restoration of mast cells above baseline levels by 24 hours post-wounding (p<0.05), likely representative of an influx of mast cells in response to localized mast cell activation (Fig. 1). In contrast, mast cells from wounds in mice injected with DSCG treatment remained granulated. These data confirm that DSCG inhibits mast cell degranulation in our model system.

### DSCG treatment leads to improved collagen fiber organization in wounds

**Figure 2:**
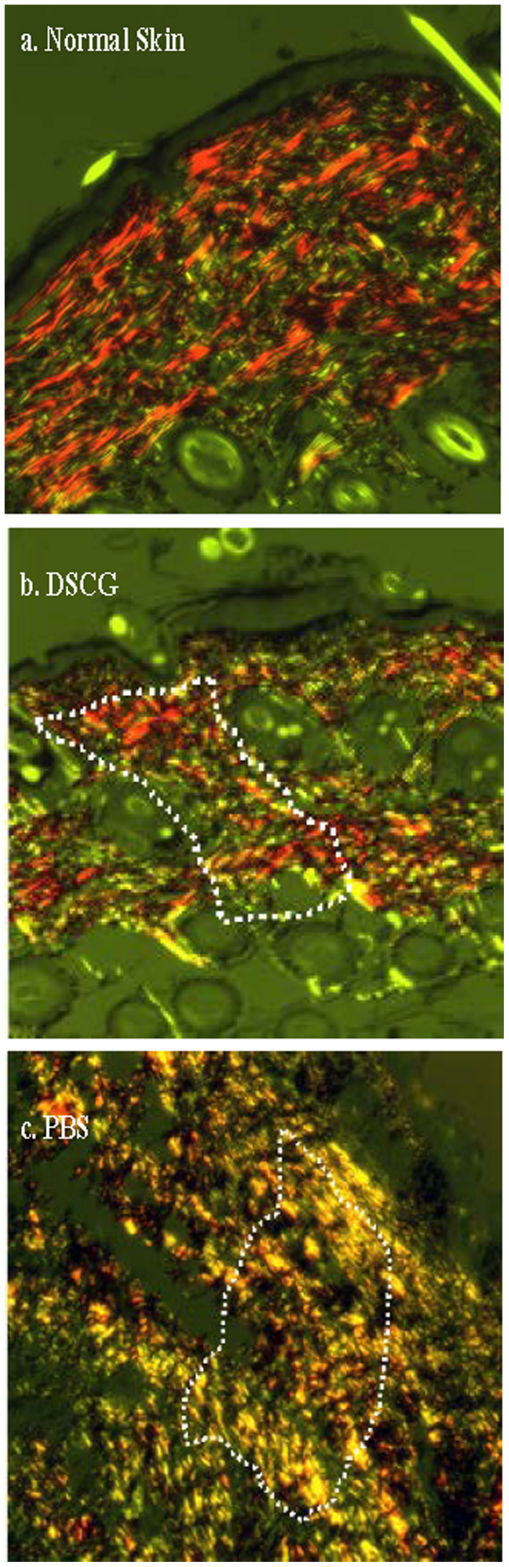
Microscopic assessment of scar tissue by Picrosirius Red staining. Sections of scar tissue from either DSCG treated mice or PBS treated mice at day 21 post-wounding were stained with picrosirius red and viewed under polarized light to detect collagen fibers. Mature collagen fibers appear red-orange and immature collagen fibers appear yellow and green. Results are representative to three independent experiments. Dot line framed areas are wounds.

The ultimate result of the repair process in adults is a scar, marked by an overproduction of disorganized collagen patching the break in the tissue. To determine the role of mast cell activation on collagen organization after wound healing, wounds from mice treated with DSCG or PBS were examined by picrosirius red staining at day 21 post-wounding and examined under polarized light. In normal skin, collagen is arranged in organized bundles that appear as brightly birefringent structures throughout the whole microscopic field. When collagen fibers are not regularly arranged, they are weakly birefringent [29]. Examination of picrosirius red stained sections of normal unwounded skin showed a dermal architecture characterized by collagen fibers with a red-orange birefringence organized in a basket weave pattern (Fig. 2a). Collagen fibers in scar tissue from mice treated with DSCG had a predominantly red-orange birefringence and were oriented in a more orderly fashion, similar to normal tissue architecture (Fig. 2b). Analysis of scar tissue from PBS-treated mice demonstrated that collagen fibers were characterized by thin, weakly birefringent yellow-green fibers with irregular collagen organization and poorly defined structure (Fig. 2c).

### DSCG treatment yields increased collagen fibrillar density in wounds

**Figure 3:**
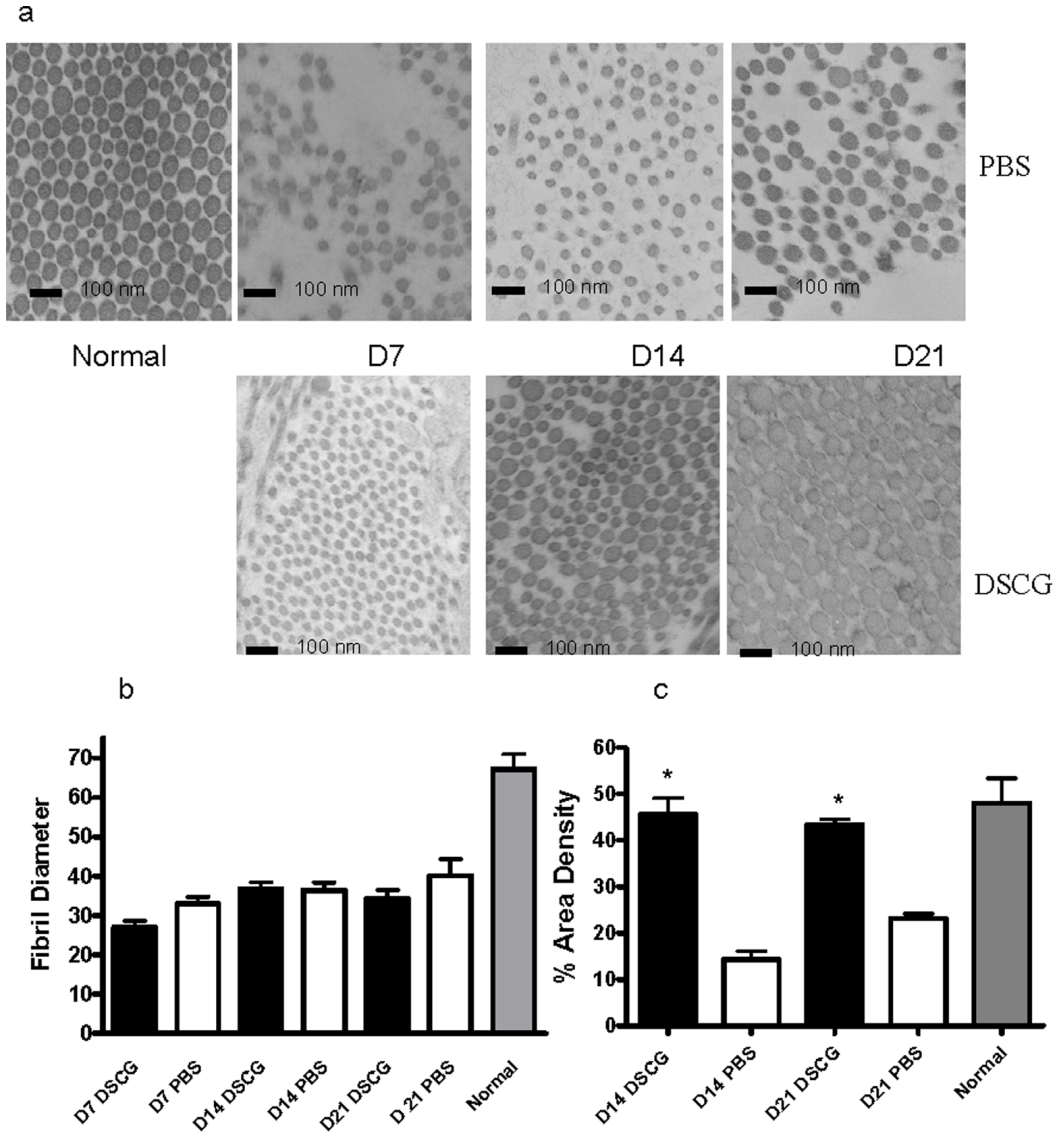
Ultrastructural analysis of collagen fibril content. (a) Transmission Electron Microscopy of normal skin and the tongue. n = 5 for all groups. TEM was performed to examine collagen fibril structure in the dermis of control mice (upper panels) and mice treated with DSCG (lower panels). (b). The diameter of individual collagen fibrils was determined for between 1100 and 2100 fibrils per section. Two separate wound sections per mouse were examined and four micrographs per mouse were analyzed. The mean fibril diameter per mouse was calculated. The average collagen diameter (n = 5) at each time point is shown. (c). Measurement of fibrillar density after DSCG treatment. The mean area density was determined by image analysis of TEM sections of wounds from either DSCG or PBS treated mice. The average area density was calculated from 10 micrographs per mouse. Data are expressed as mean ± SEM (n = 5). *p<0.01 by t-test.

The results from the picrosirius red staining indicated that treatment with DSCG prior to wounding resulted in tissue similar to normal skin. To analyze the architecture of the skin in mice treated with DSCG compared to control mice, we used TEM to measure the diameter of fibrils in scar tissue at 7, 14 and 21 days post-wounding. Changes in the homogeneity of fibril diameter correlate to fibrosis and scarring. The representative photographs in Fig. 3a show that compared to unwounded skin, both treatment groups show an increase in small diameter fibrils, suggesting scar formation. However, there was no significant difference in the distribution of fibril diameter (Fig. 3b). While the fibril diameter was unchanged, fibril density was influenced by inhibition of mast cells. Comparison of fibril density between the two treatment groups revealed that wounds from DSCG treated mice exhibited a much higher fibrillar density (Fig. 3c).

### DSCG treatment causes a reduction in wound scar width

**Figure 4:**
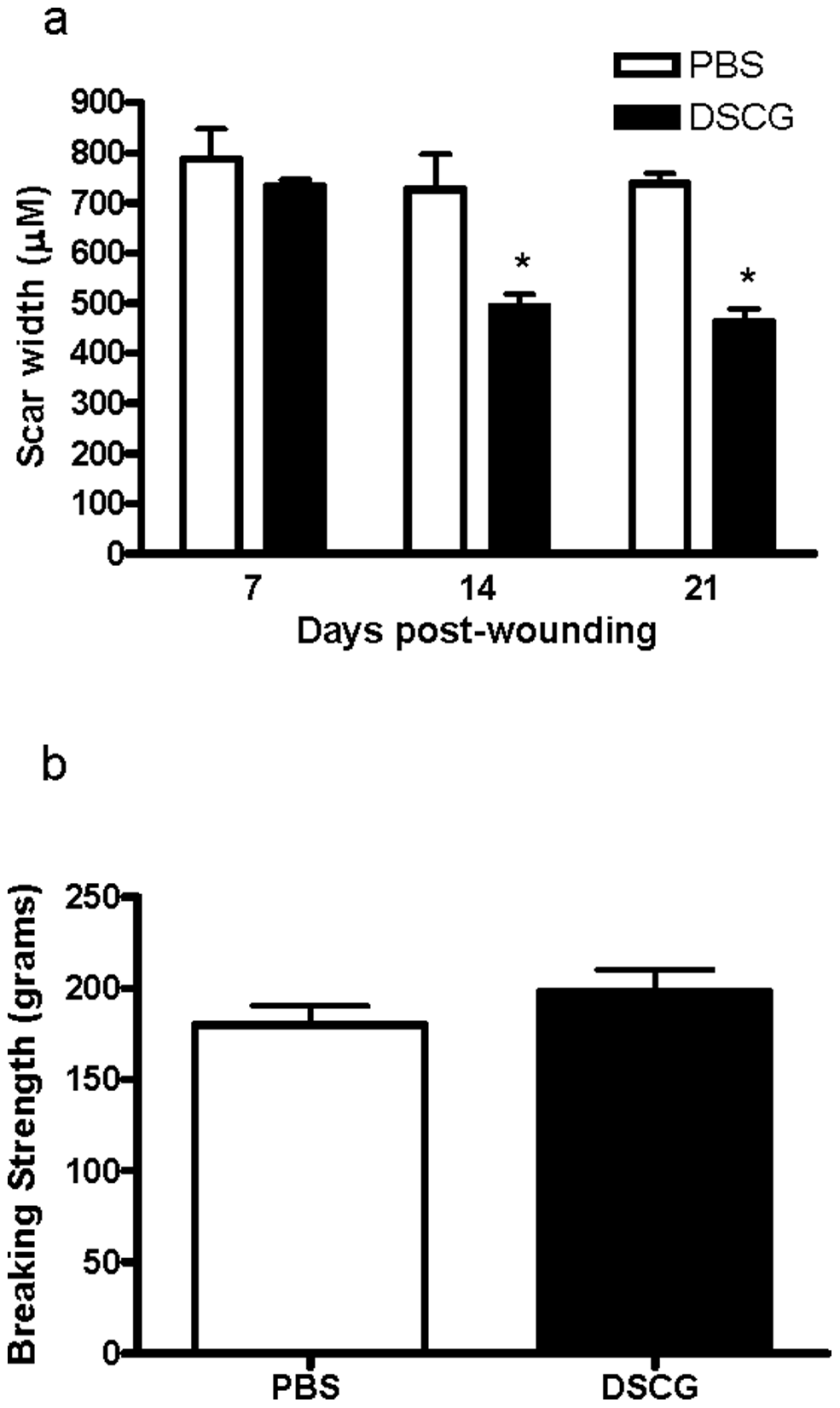
Effect of mast cell inhibition with DSCG on scar width and wound breaking strength. (a) Average scar width in mice treated with DSCG compared to PBS treated mice. H & E staining was used to measure scar with at 7, 14 and 21 days post-wounding in DSCG treated mice (black bars) and control mice (white bars). N = 5. *p<0.05. (b) Effect of DSCG treatment on wound breaking strength. Incisional wounds were prepared on the back of mice treated with DSCG (black bar) or PBS (white bar). N = 10 for both groups. Wounds were excised at day 14, and two skin strips per mouse were used from the upper and lower back of the mouse. Both strips were subjected to tensiometric analysis. The bars indicate the means of wound breaking strength in grams.

Another measurement of healing capability is the assessment scar width. To determine if DSCG treatment and decreased mast cell degranulation influenced scar width, we measured the width of wound sections from both PBS and DSCG treated animals. Scar width was significantly reduced in mice treated with DSCG, suggesting that blockade of mast cell activation reduces cutaneous scarring (Fig. 4a).

### DSCG treatment does not affect wound breaking strength

The success of dermal healing can be measured by assessing the ability of healing skin to withstand tensile forces. To determine if wounds from DSCG treated mice were equally strong as control wounds, we measured wound breaking strength. As shown in Fig. 4b, there was no significant difference in wound breaking strength in mice treated with DSCG compared to mice treated with PBS. These results suggest that while DSCG does reduce scarring, this treatment does not decrease the strength of the healed skin.

### DSCG treatment causes decreased wound inflammation

**Figure 5:**
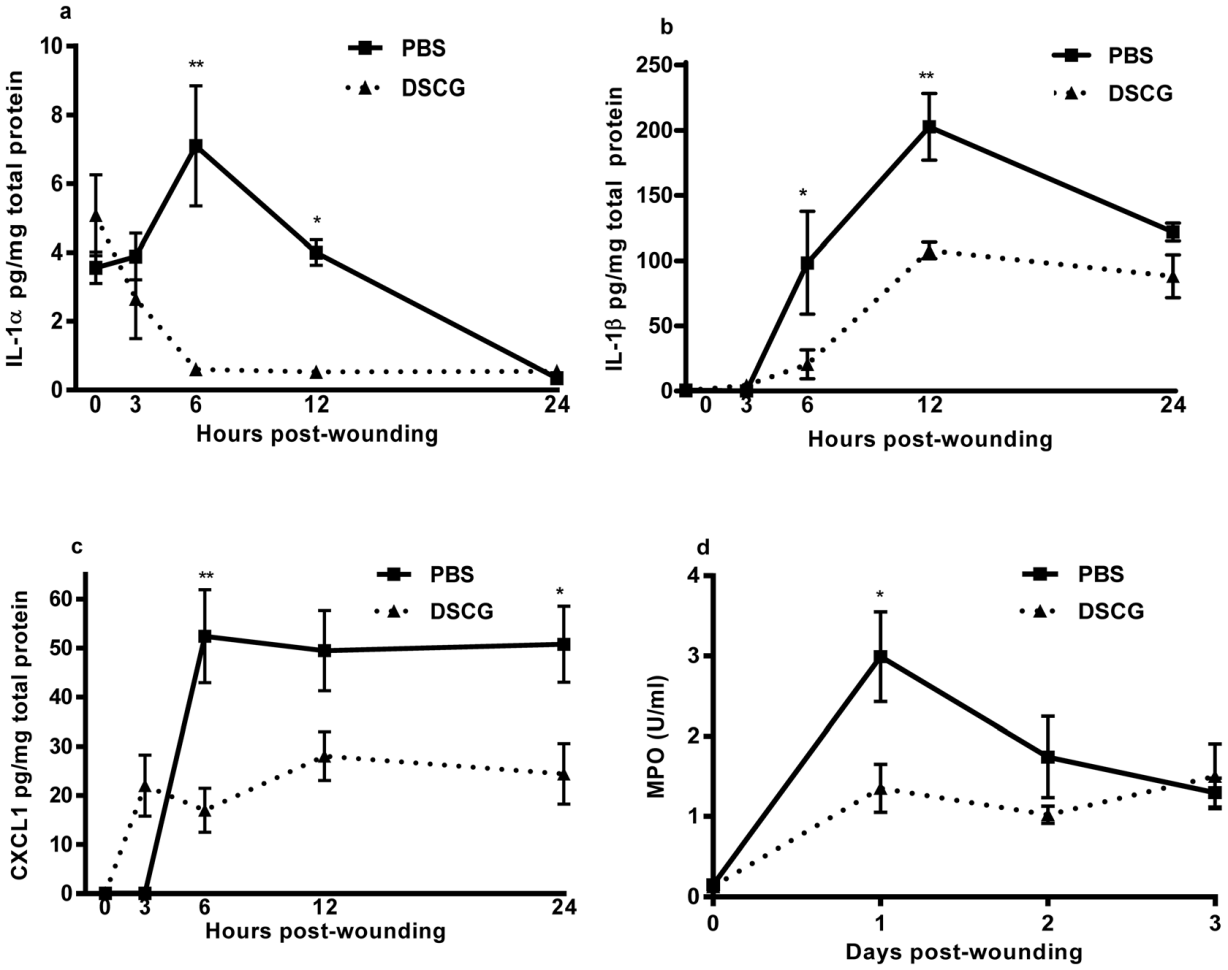
Effect of inhibition of mast cells with DSCG on wound cytokine and neutrophil MPO levels. (a) IL-1α, (b) IL-1β, (c) CXCL1 protein levels, and (d) MPO activity were measured by ELISA in wound homogenates d from mice treated with DSCG or PBS. Results are expressed as the mean ± SEM (n = 3). * p<0.05 ** p<0.01 by 2-way ANOVA followed by a Bonferroni post-test.

The granules stored within mast cells contain a multitude of preformed inflammatory mediators. Upon activation, the granules are released into the surrounding tissue, leading to the initiation if inflammation. To determine if the prevention of mast cell degranulation alters the inflammatory response, levels of IL-1α, IL-1β and CXCL1 were measured at 3,6, 12 and 24 hours post-wounding. Levels of IL-1α in unwounded skin were similar in both treatment groups. In mice treated with PBS, IL-α production peaked at 6 hours post-wounding and gradually decreased, while levels of IL-1α in mice treated with DSCG were significantly decreased at 3,6 and 12 hours post-wounding (Fig. 5a). Levels of IL-1β in unwounded skin of both groups were similar. While both groups showed a peak of IL-1β production at 6 hours post-wounding, there was a significant decrease in the IL-β production in wounds of mice treated with DSCG (Fig. 5b). Similarly, CXCL1 (murine IL-8) levels peaked by 12 hours in both groups, but CXCL1 production was significantly decreased in mice treated with DSCG at 6, 12 and 24 hours post-wounding (Fig. 5c). These data are consistent with the idea that mast cell degranulation initiates the production and secretion of early inflammatory cytokines and further demonstrate the efficacy of DSCG treatment decreasing inflammation in response to wounding.

The reduction in early inflammatory cytokines in wounds from DSCG treated mice suggested that inflammatory cell recruitment might also be affected by reducing mast cell activation. To determine if reduction in inflammatory cytokines after DSCG treatment reduced neutrophil infiltration, wounds were subjected to MPO analysis. MPO activity in DSCG treated mice was significantly decreased in response to injury (Fig. 5d) especially at day 1 after treatment (p<0.05).

### DSCG treatment does not affect wound re-epithelialization

**Figure 6:**
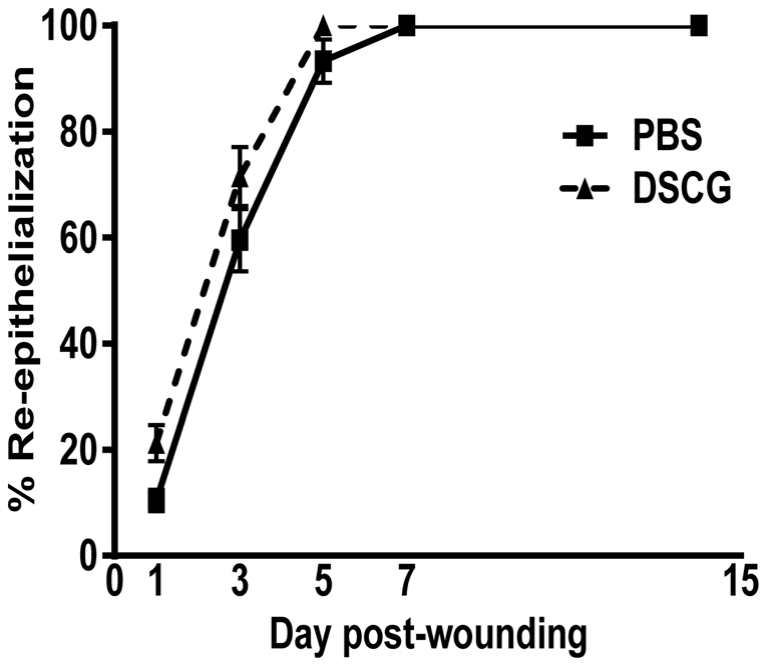
Re-epithelialization of wounds in DSCG treated mice. The rate of re-epithelialization was measured by histomorphometric analysis of tissue sections from the central portion of the wound.

To determine if mast cell inhibition affects wound closure, the rate of re-epithelialization was measured at days 3, 5 and 7 days post-wounding in DSCG and control mice. DSCG treatment had no effect on the process of wound re-epithelialization. Wound closure occurred at a similar rate in mice treated with DSCG and PBS (Fig. 6).

### Tryptase β1 promotes differentiation of fibroblasts to myofibroblasts and induces collagen I mRNA expression

**Figure 7:**
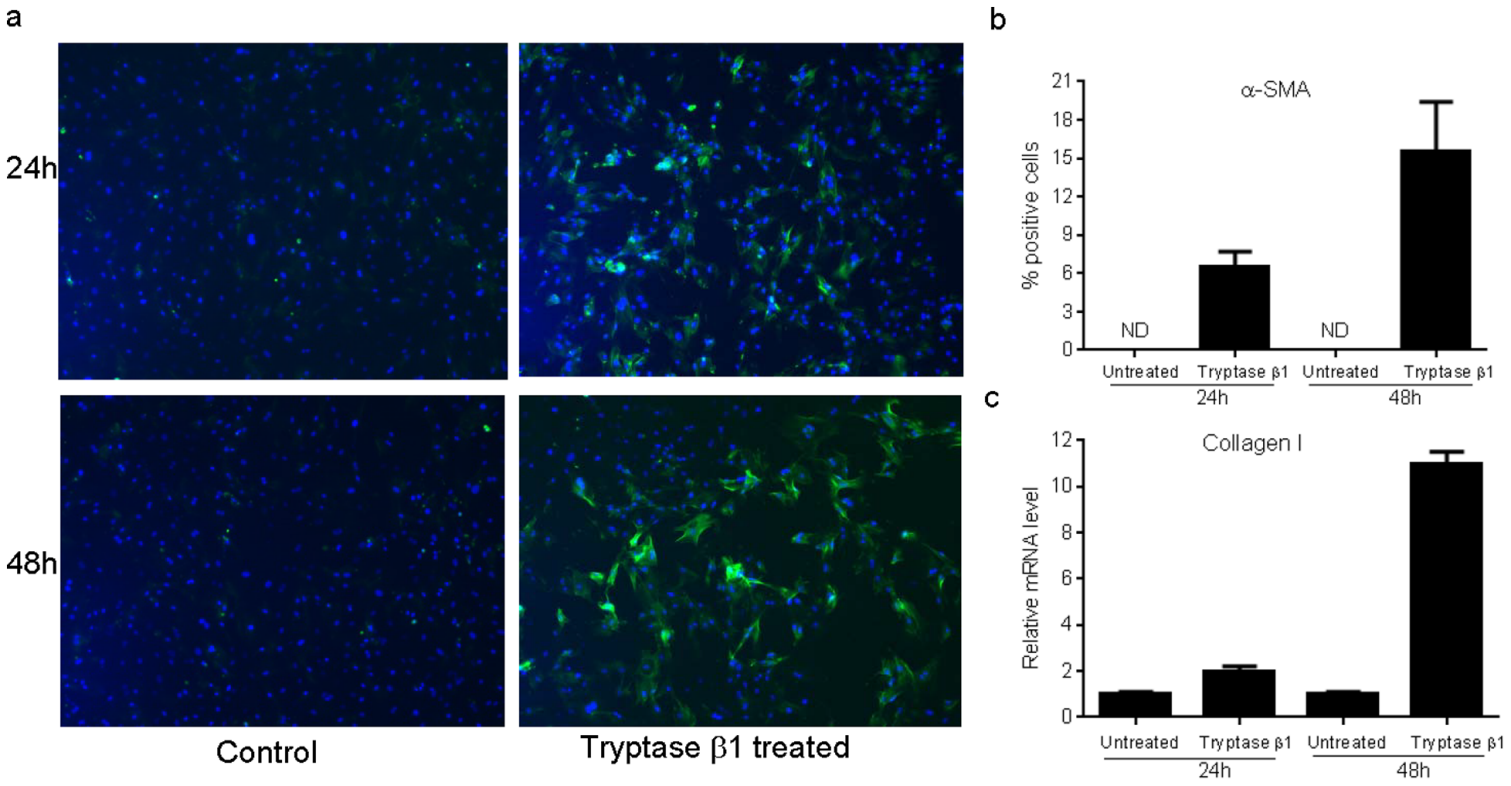
Tryptase β1 induces α-SMA and collagen I expression in dermal fibroblasts. (a). Tryptase β1 induces differentiation of dermal fibroblasts into myofibroblasts. Dermal fibroblasts were treated with tryptase β1 for 72 hours and α-SMA expression was detected using direct immunofluorescence. Green stained cells are α-SMA positive myofibroblasts. DAPI was used to counterstain nuclei (blue). (b). Percent of α-SMA positive myofibroblasts. ND: not detectable. (c). Relative mRNA expression of collagen I 24 hours after tryptase β1 treatment determined by real time PCR. Results were the averages of triplicate wells.

Previous studies have demonstrated an association of mast cells with scar formation and fibrosis, conditions typified by the presence of myofibroblasts. To examine whether mast cell tryptase might influence fibroblast function, we treated fibroblasts with tryptase and examined them for the production of α-SMA, a marker of myofibroblasts. Tryptase β1 induced 6.5+1.2% and 15.5+3.9% of dermal fibroblasts to become α-SMA positive myofibroblasts at 24 and 48 hours after treatment respectively (Fig. 7a&b). No α-SMA positive cells were observed in control cultures not treated with tryptase β1 at either time point (Fig. 7a). Furthermore, dermal fibroblasts expressed significantly more collagen I mRNA after being treated by tryptase β1. When compared to control, levels of collagen I mRNA were 11 fold higher in treated cells after 48 hours of exposure to tryptase, (Fig. 7c). These results are consistent with previous reports using human fibroblasts [8]–[10], [30].

### Levels of mast cell derived tryptase increase in healing wounds

**Figure 8:**
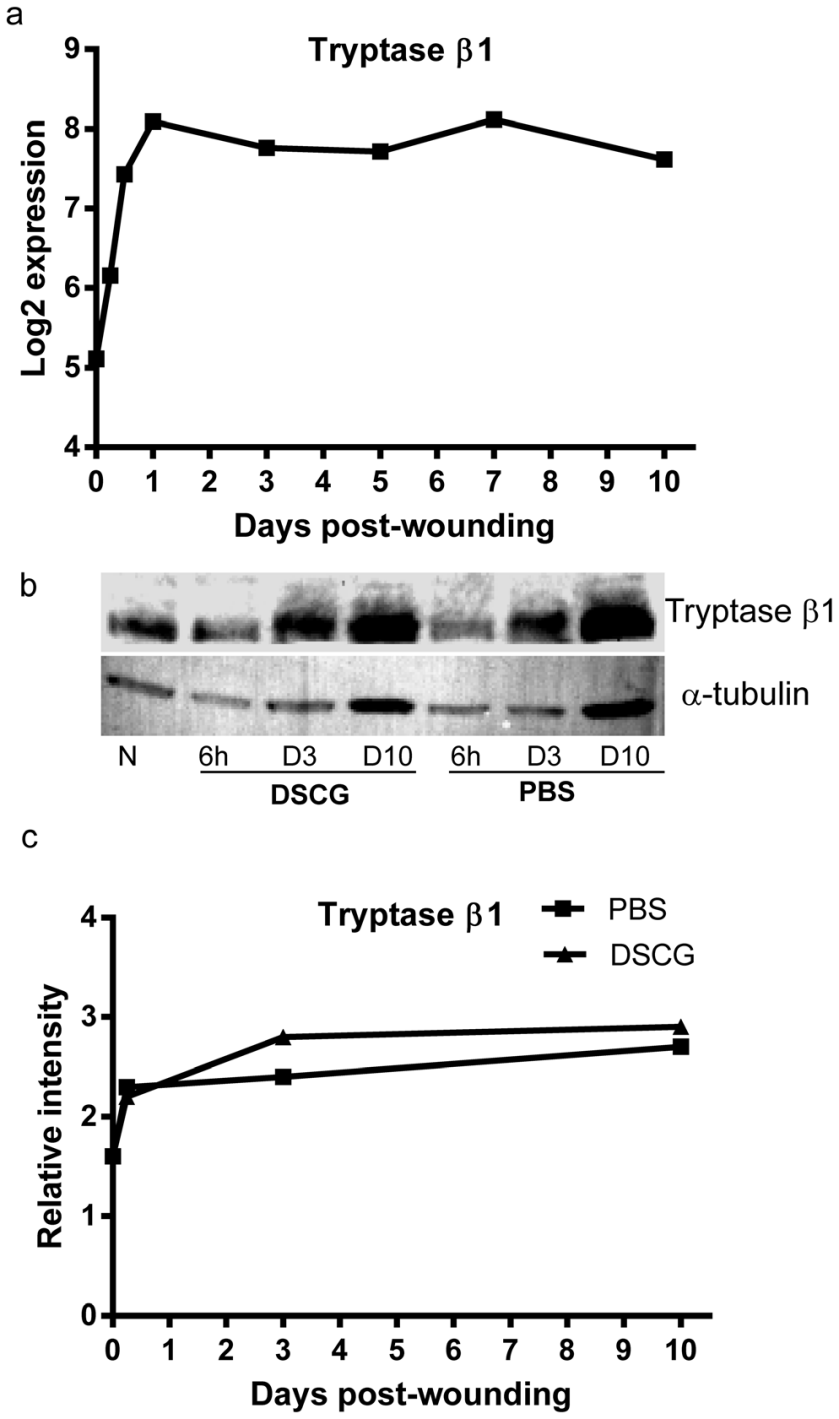
Tryptase β1 expression in skin wounds. (a). Tryptase β1 transcript levels during the course of wound healing were determined by microarray analysis [31]. Tryptase β1 gene expression significantly increased started at 6 hours after wounding, and remained increased through day 10 (p<0.05 by a one –way ANOVA test, n = 3 at each time points). (b). Western blot analysis of the protein levels of tryptase β1 in skin wounds after DSCG or PBS treatment. α-tubulin was used as a protein loading control. (c). Relative intensities of the bands shown in (b) after being normalized to α-tubulin. N: normal skin.

An analysis of the microarray database from murine wounds demonstrated that tryptase β1 transcript is significantly upregulated in the healing wound [31]. Levels of tryptase β1 gene expression started to increase at 6 hours after wounding and reached peak levels at 24 hours after injury (Fig. 8a). Interestingly, a high level of expression was sustained from 24 hours to 10 days after wounding (Fig. 8a). Immunoblot analysis of wound lysates demonstrated that the protein levels of tryptase β1 in wounds (Fig. 8b&c) showed a similar pattern to the gene expression levels (Fig. 8a). However, there were no significant changes observed in tryptase β1 levels between the wounds of DSCG treated and control mice (Fig. 8b&c).

## Discussion

In the present study, we show that the inhibition of mast cell degranulation during early wound repair reduces later scar formation without disrupting wound breaking strength. Moreover, the data demonstrate that DSCG treatment reduces the immediate inflammatory response in wounds.

Given that the inflammatory phase of wound healing promotes downstream healing processes and that the amount of inflammation can affect the remodeling phase, we speculated that mast cell blockade could alter scar formation. Two recent studies have examined the epidermal components of wound healing using the WBB6F1-kit^w^/kit^w-v^ mice. While Egozi et al found no changes in re-epithelialization in mast cell deficient mice [15], Weller et al observed an initial change in re-epithelialization that reached normal levels by day 10 post-wounding [32]. Mast cell reconstitution alleviated the early differences, but did not increase re-epithelialization beyond control animals. The authors suggested that the initial enhanced re-epithelialization could be due to the absence of histamine stored within mast cells which could promote cutaneous repair acting as a keratinocyte mitogen [32]. While both of these studies suggest a role for mast cells using the kit^w^/kit^w-v^ mouse, these mice have other deficiencies such as minor alterations in immune cell generation in addition to anemia, both of which could alter the wound healing response [17], [18]. Therefore, we used a pharmacological approach to inhibit mast cell degranulation.

The predominant step in the sequence of biochemical events following immunological stimulus of mast cells is a transient increase in the permeability of the plasma membrane to calcium ions [20], [22], [33]. DSCG inhibits mast cell degranulation and has an established function in the prophylactic treatment of allergic diseases [25], [34]. Although the precise molecular mechanism of inhibition is unknown, studies have shown that DSCG bind s specifically to the external membranes of mast cells and basophils in a calcium-dependent fashion. Binding of these channels is believed to stabilize mast cell granules, prevention degranulation [25], [34]. These studies suggest that inhibitory effects of DSCG on mast cell make it an interesting candidate for the treatment of wounds.

Previous studies by us and others that utilized the WBB6F1-kit^w^/kit^w-v^ mice were limited to the inflammatory and proliferative stages of wound healing, but did not examine scar formation. Since DSCG treatment did not change the rate of wound re-epithelialization, we performed additional analysis of the later remodeling and scar formation aspects of repair. These studies revealed that inhibition of mast cell degranulation with DSCG resulted in a wound bed with more organized collagen, and with architecture more similar to normal skin. An ultrastructural level analysis of fibril diameter showed that mast cell inhibition did not seem to impact this parameter. However, the fibrillar density in wounds of mice subjected to DSCG was much greater than those of control mice. Despite the increase in fibrillar density, DSCG treatment did not improve wound breaking strength. This may not be surprising as multiple studies suggest that it is a challenge to improve wound breaking strength of normal animals.

Of great interest was the finding that the inhibition of mast cells led to a significantly reduced scar width. This finding suggests an important effect of mast cells in wound healing and scar development that might be eventually exploited to improve healing outcomes. Our results suggest that this effect may involve both direct and indirect mechanisms. The indirect mechanism may derive from the reduction in wound inflammation that was seen following the inhibition of mast cells.

In our current study, the number of mast cells in 12 hour-wounds was significantly decreased in the PBS control group but remained steady in DSCG treated mice. The reasons for this decrease might be massive degranulation, apoptotic death and/or migration away from the wounds. However, previous studies demonstrate that DSCG had strong inhibitory effect of mast cell degranulation [25], [34]. Therefore, it seems very likely that the steady levels of mast cells in the wounds of DSCG treated animals result from the inhibition of degranulation.

A correlation between inflammation and fibrosis in response to wound healing has been clearly demonstrated in the fetal wound model and further supported by our own studies of reduced scaring during mucosal healing [23], [27], [35]. Of the many factors that contribute to privileged healing, a decreased inflammatory response appears to be very important. A direct mechanism for mast cell influence on scar formation was suggested by our studies of mast cell tryptase. Our results demonstrate that tryptase levels are high in wounds for an extended period of time. Similar to studies in other systems, we found that tryptase can induce murine fibroblasts to adopt the myofibroblast phenotype. Together, these data suggest that mast cell tryptase may affect scar formation. Thus, the reduction in scar formation in DSCG treated mice may be due to the fact that this drug inhibits mast cell degranuation and extracellular release of tryptase.

To assess whether DSCG treatment does in fact reduce active tryptase levels, we performed immunoblot analysis of tryptase within wounds of DSCG mice. Interestingly, this experiment demonstrated that the levels of tryptase in wounds of DSCG treated mice were not significantly different than control. In considering this result, we realized that this negative finding is most probably due to the fact that Western blot, the assay used in current study, cannot distinguish between free tryptase that is released by mast cells and tryptase localized within mast cells. Because DSCG does not delete but only stabilizes mast cells, tryptase remains in the cells and is detectable, although not active in the wounds of DSCG treated mice.

We do not know if DSCG has any direct effects on the recruitment or functions of neutrophils. In our study we found that MPO levels were significantly reduced in the DSCG treated wounds (24 hours), which is consistent with recent studies that observed DSCG treatment could decrease the influx of neutrophils in bronchoalveolar lavage fluid or peritoneal cavity [36].

One aspect of wound repair that was not investigated in the current study is the influence of DSCG treatment on wound angiogenesis. Mast cells produce proangiogenic mediators such as fibroblast growth factor-2 and vascular endothelial cell growth factor (VEGF) [14], [37], [38], suggesting that mast cells may play a role in angiogenesis. However, a previous study from our lab demonstrated that that wounds of WBB6F1-kit^w^/kit^w-v^ mast cell deficient and control mice have similar blood vessel content and VEGF levels [15]. Although the current study employs a different method of interfering with mast cell function, our prior result in the WBB6F1-kit^w^/kit^wv^ mast cell deficient mice suggests that DSCG treatment would not alter wound angiogenesis.

The experiments presented here provide evidence that the mast cell, once thought to only play a role in allergic responses, could be a key component of inflammatory initiation in response to wound healing. The concept that inhibition of mast cells during wound healing leads to more organized collagen architecture and smaller scars without disruption of wound integrity implicates mast cells as a therapeutic target. This could be especially true in conditions characterized by excess scarring. Further understanding of the mechanism of reduced scar formation as a result of mast cell inhibition may allow the development of applications to promote optimal wound healing.
